# Fungemia caused by *Penicillium marneffei* in an immunocompetent patient with COPD: A unique case report

**DOI:** 10.1097/MD.0000000000009658

**Published:** 2018-01-19

**Authors:** Xiaoming Yu, Xueding Cai, Xiaomei Xu, Lin Zhang, Xiaoying Huang, Liangxing Wang, Yanfan Chen

**Affiliations:** a Division of Pulmonary Medicine, The People's Hospital of Cangnan, Wenzhou Medical University, Cangnan; b Division of Pulmonary Medicine, The First Affiliated Hospital of Wenzhou Medical University, Wenzhou, Zhejiang, China.

**Keywords:** COPD, fungemia, *Penicillium marneffei*, voriconazole

## Abstract

**Rationale:**

This report describes a rare case in Wenzhou city of Zhejiang province that a non-HIV infected male recovering from fungemia caused by *Penicillium marneffei* (*P. marneffei*). Interestingly, it's very easy to misdiagnose with aspergillosis, a fungal disease prevalent in Wenzhou, during the whole procedure.

**Patient concerns:**

An 80-year-old Chinese male subject with pre-existing chronic obstructive pulmonary disease (COPD) presented with symptoms of chest tightness and high fever for a month.

**Diagnoses:**

Fungal culture from the blood isolated *P marneffei*. Naturally, the patient was diagnosed with *P marneffei* fungemia. However, he was proven serologically to be negative for human immunodeficiency virus (HIV).

**Interventions:**

The patient was treated with voriconazole at 200mg/dL every 12 hours via intravenous administration.

**Outcomes:**

The fever returned to normal and chest tightness disappeared gradually after a week of voriconazole treatment.

**Lessons:**

A high level of clinical suspicion and awareness is necessary for early diagnosis of *P marneffei* fungemia, especially in elder patients with underlying diseases.

## 1 Introduction

*Penicillium marneffei* is a thermical dimorphic fungus that causes progressive and fatal fungal infection, especially in human immunodeficiency virus (HIV)-infected patients. Diagnosis of *P marneffei* fungemia was made by examination of a peripheral blood smear or fungal culture from blood in HIV-infected patients.^[1–3]^ To the best of our knowledge, no *P marneffei* fungemia cases have been reported in immunocompetent individuals. Moreover, patients living with chronic obstructive pulmonary disease (COPD) rarely suffered from infection caused by *P marneffei*. However, an immunocompetent French traveler with COPD was reported to be infected with *P marneffei* from bronchoalveolar lavage (BAL) culture in 2014.^[4]^ Different from the case just mentioned, here we reported a unique case that the non-HIV infected patient who had a history of COPD was diagnosed with *P marneffei* fungemia after analysis of fungal culture from blood.

## 2 Case report

**Figure 1:**
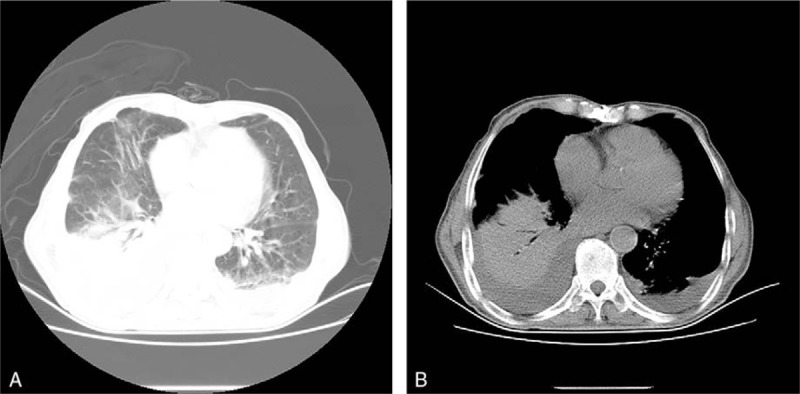
A, B, CT scan of the lung showing (A) bilateral compressive atelectasis with large pleural effusions in lung window and (B) bilateral large layering pleural effusions in mediastinal window.

**Figure 2:**
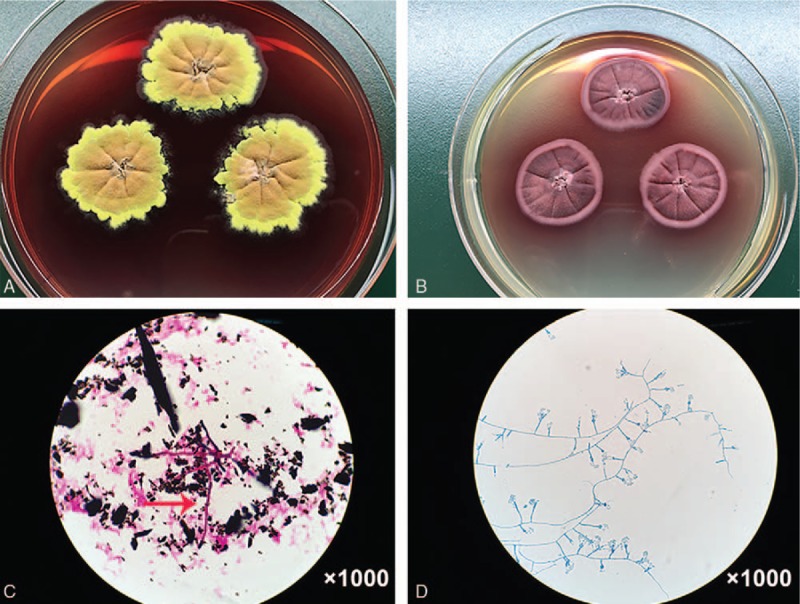
A, B, C, D fungal culture from blood showing (A) fried egg-like mold colony of *P marneffei* with a red diffusible pigment on Sabouraud Dextrose agar (after 10 days incubation at 25 °C) and (B) yeast colony of *P marneffei* (after 10 days incubation at 35 °C). The mold was smeared for Gram staining from blood culture showing (C) the red and rod-shaped hyphae (red arrow). Lacto-phenol cotton blue stain of *P marneffei* revealed (D) paintbrush-like clusters hyphae and conidiophores.

A southeast Chinese man in his 80s presented with a 1-month history of chest tightness and intermittent high fever. The patient with pre-existing COPD had surgeries done on mediastinal tumor and deep venous thrombosis (DVT) 2 years ago before admission. But the patient could not provide detailed information about his mediastinal tumor. He was referred to our clinic under the suspicion of pulmonary infection. The vital signs were *T* 38.6 °C, BP 145/75 mm Hg, heart rate 106 beats per minute, and respiratory rate 20 breaths per minute during the initial examination. A physical examination was unremarkable except for the visible mold in the oral mucosa. Chest computed tomography (CT) showed bilateral lung scattered inflammatory infiltration and bilateral pleural effusions (Fig. 1). No bronchial abnormality was noted by fiber bronchoscope. Bone marrow biopsy showed no abnormal cells. Initial laboratory routine examinations were shown in Table 1. Diagnosis of bacterial infection was first consideration based on the clinical characteristics and auxiliary examinations. Imipenem cilastatin was promptly used on his admission. However, the antifungal therapy was adopted instead of empiric antibacterial therapy when the outcomes of serum (1 → 3)-β-D-glucan antigen assay (BG assay) and serum aspergillus galactomannan antigen assay (GM assay) were positive and simultaneously, the outcome of antibacterial treatment was ineffective. It was highly likely that the patient was infected with aspergillosis endemic in the southeast China. The patient was treated with voriconazole at 200 mg/dL every 12 hours via intravenous administration starting from January 24, 2017. Unexpectedly, the blood cultures were positive for *P marneffei*, a rare dimorphic fungus which usually causes a fatal infection among HIV-infected individuals (Fig. 2). The fever returned to normal and chest tightness disappeared gradually after a week of voriconazole treatment. Taken the fungal culture and the effective therapy into consideration, the patient was diagnosed with *P marneffei* fungemia. He received the follow-up antifungal treatment and assessment.

**Table 1:**
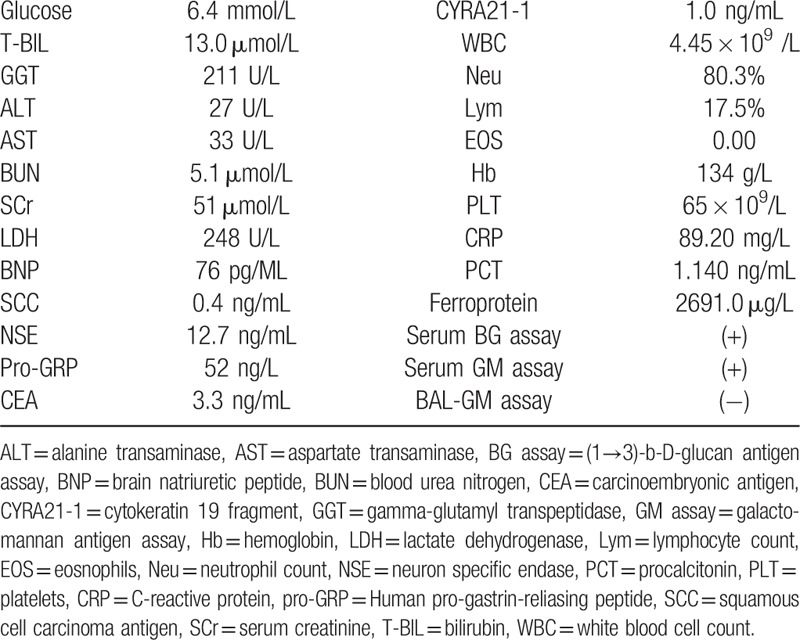
Laboratory findings at first presentation.

## 3 Discussion

*P marneffei*, originally isolated from a bamboo rat, *Rhizomys sinensis*, is an emerging pathogenic fungus.^[5]^ The fungal infection caused by *P marneffei* is an AIDS-defining illness and, next to tuberculosis and cryptococcosis, the third most common opportunistic infection in AIDS patients in tropical Southeast Asia.^[6]^ Since the first case was reported in 1998 that an AIDS patient infected with fungemia by *P marneffei*,^[7]^ an increasing number of *P marneffei* fungemia cases have been reported in HIV-infected individuals recently.^[3,8–10]^ However, *P marneffei* fungemia is a rarely reported diagnosis in an HIV-negative individual with a history of COPD. In this article, we present the first such case worldwide.

Clinical manifestations of *P marneffei* infection are nonspecific; it usually presents with fever, weight loss, skin lesions, generalized lymphadenopathy, hepatomegaly, and respiratory signs.^[11]^ In this case, the patient presented with fever and chest tightness, no other special clinical manifestations. He was immunocompetent, but his immunity was probably impaired due to long-standing COPD, mediastinal tumor resection and aging body. Besides the infection, malignant lymphoma is not entirely excluded due to the aging body, fever, and surgical history of mediastinal tumor. Nevertheless, the proofs that there was no generalized superficial lymphadenopathy and no abnormal cells in bone marrow biopsy provided nonsupport for this disease. The patient was misdiagnosed with invasive aspergillosis as a clinical diagnosis because, (1) the long-term use of antibiotics and glucocorticoid for COPD-defining infection control, (2) symptoms persisted after antibacterial treatment, and (3), positive results of BG assay and GM assay were very similar to characteristics of invasive aspergillosis, which is a common disease in southeast China. However, we ignored the fact that galactomannan also exists in the cytoderm of *P marneffei* which leads to the positive result of GM assay. Fortunately, voriconazole is one of the effective, well-tolerated therapeutic options for this disease.^[12]^*P marneffei* has a high risk of relapse, so it is necessary to follow-up of the patient and evaluate any long-term effects on him.

The nonspecific presentation and high mortality rate of *P marneffei* infection highlight the importance of a rapid diagnosis and treatment, especially for the severe *P marneffei* fungemia. The most widely used antifungal drugs for the treatment of *P marneffei* infection are amphotericin B and itraconazole. Itraconazole is more widely adopted in the therapy as the predictable nephrotoxicity of amphotericin B limits its usage. Voriconazole is a new extended-spectrum triazole antifungnal agent with potent in vitro activity against *P marneffei* which is superior to that of amphotericin B and itraconazole.^[13]^ For our patient, he was misdiagnosed with aspergillosis at first. Fortunately, voriconazole is an effective drug in the treatment of disseminated *P marneffei* infection as reported by a retrospective study.^[12]^

## 4 Conclusions

*P marneffei* is an AIDS-defining endemic mycosis in Southeast Asia. Herein, we describe the first case to our knowledge of *P marneffei* fungemia in an HIV-negative individual with pre-existing COPD. Furthermore, among immunocompetent individuals, clinicians should be aware of *P marneffei* fungemia in aging patients with underlying diseases and surgical histories.

Authors’ contributions: YFC and XMY conceived the study and developed the search strategy. XMY conducted the review of relevant articles, and produced the draft of the manuscript. XDC and XMX contributed to the diagnosis and treatment. LZ participated in data collection and interpretation. XYH and LXW reviewed and edited the manuscript. All authors read and approved the final manuscript.
